# Hospital staff participation in a national hip fracture audit: facilitators and barriers

**DOI:** 10.1007/s11657-019-0652-8

**Published:** 2019-11-21

**Authors:** Stijn C. Voeten, Leti van Bodegom-Vos, J. H. Hegeman, Michel W.J.M. Wouters, Pieta Krijnen, Inger B. Schipper

**Affiliations:** 1 Department of Trauma Surgery, Leiden University Medical Center, Albinusdreef 2, 2333ZA, Leiden, The Netherlands; 2 Dutch Institute for Clinical Auditing, Leiden, The Netherlands; 3 Department of Biomedical Data Sciences, Leiden University Medical Center, Leiden, The Netherlands; 4 Department of Trauma Surgery, Ziekenhuisgroep Twente, Almelo-Hengelo, The Netherlands; 5 Department of Surgery, Netherlands Cancer Institute-Antoni van Leeuwenhoek, Amsterdam, The Netherlands

**Keywords:** Clinical audit, Implementation science, Hip fractures

## Abstract

***Summary*:**

To ensure meaningful results in a clinical audit, as many hospitals as possible should participate. To optimise participation, the data collection process should either be performed by additional staff or be automated. Active participation may be promoted by offering relevant external parties insight into the actual quality of care.

**Purpose:**

The aim of the study was to identify which facilitators and barriers experienced by hospital staff are associated with participation in the ongoing nationwide multidisciplinary Dutch Hip Fracture Audit (DHFA).

**Methods:**

A survey including questions about the respondents’ characteristics, hospital level of participation and factors of influence on DHFA participation was sent to hip fracture surgeons. The factors were based on results of semi-structured interviews held with hospital staff involved in hip fracture care. Univariable and multivariable logistic regression analyses were used to establish which respondent characteristics and factors were associated with participation and active participation (≥ 80% of patients registered) in the DHFA. Factors significantly increasing the (active) participation in the DHFA were classified as facilitators, and factors significantly decreasing the (active) participation in the DHFA as barriers.

**Results:**

One hundred nine surgeons filled out the questionnaire. The factors most agreed on were availability of staffing capacity for data collection and automated data import. A lower intention to participate was associated with being an academic surgeon (odds ratio, 0.15; 95% confidence interval, 0.04–0.52) and an orthopaedic surgeon (odds ratio, 0.30; 95% confidence interval, 0.10–0.90). Data sharing with relevant external parties was associated with active participation (odds ratio, 3.19; 95% confidence interval, 1.14–8.95).

**Conclusions:**

To improve participation in a nationwide clinical audit, it seems that the data collection should either be performed by additional staff or be automated. Active participation is facilitated if audit data is made available to other parties, such as insurers, healthcare authorities or policymakers.

## Introduction

In a clinical audit, prospective data of the treatment process and of patient outcomes is collected in a systematic way. Based on these data, feedback is given to provide hospitals insight into their own performances. Also, in a clinical audit, hospitals are benchmarked on pre-established quality indicators to define best practices that can serve as examples for underperforming hospitals. The aim of this continuous process is that hospitals implement changes in the treatment process based on the feedback, with the ultimate goal to improve the quality of care [1].

In the last decade, audits for different diseases have been initiated [2–8]. The participation in an audit can be a major logistic challenge to hospitals. Active participation may involve a high registration load because data recorded in the electronic health record (EHR) often has to be copied into a separate clinical audit database. This implies incorporation of the data entry process into routine hospital working procedures. To be able to deliver meaningful feedback to hospitals and for an audit to be successful in improving quality of care, it is important that as many hospitals as possible participate and that the data registry is as complete as possible. In order to ensure the successful implementation of new nationwide multidisciplinary clinical audits, reasons for both willingness (‘facilitators’) and refusal (‘barriers’) to participate in an audit should be clear. Knowledge about these facilitators and barriers may also offer opportunities to enhance participation in ongoing audits. In literature, potential facilitators and barriers for conducting a clinical audit are described [9, 10]. To our knowledge, evidence is lacking about which facilitators and barriers actually influence hospital participation in new and ongoing audits.

The aim of this study was to identify which factors (facilitators and barriers) experienced by hospital staff are associated with hospital participation in a nationwide multidisciplinary clinical audit on hip fracture care that was recently started in the Netherlands, the Dutch Hip Fracture Audit (DHFA).

## Methods

### Dutch Hip Fracture Audit

The DHFA is a continuous nationwide multidisciplinary hip fracture audit, which was started in April 2016 at the Dutch Institute for Clinical Auditing [8]. In the Netherlands, there are approximately 18,500 hip fracture patients each year in 81 hospitals [11]. All these hospitals were invited to participate in the DHFA. Table 1 outlines what participation in the DHFA actually involves for a hospital.

**Table 1:** What does participation in the DHFA mean at hospital level?

Subscription	- Hospital participation in the DHFA is not compulsory, but the four medical associations that jointly developed the DHFA dataset advised their members to participate. - A hospital can subscribe to participate, subject to agreement of its executive board. - Participation is free of costs. - There are no financial incentives for participation.
Data gathering	- Data from the electronic health record needs to be entered into the Dutch Hip Fracture database. - The DHFA does not provide nationwide staffing or other resources or personal for the data gathering. - It is up to the local hospital to organise the data gathering process and to decide which medical professional(s) is (are) responsible for the data gathering process. - For all hip fracture patients of 18 years or older, 45 items need to be entered into a web-based survey spread at three different moments in the treatment process. - These three different moments are on admission, 3 months after admission and 1 year after admission. - Patients having a pathologic fracture due to a malignant disease or a periprosthetic fracture should not be registered.
Advantages	- A weekly updated report to provide insight into the hospital’s own clinical performance on structures, processes and outcomes of hip fracture care - Hospitals’ clinical performances are also benchmarked. This can help hospitals determine how their treatment process can be optimised, and how specific intervention can be implemented to ensure a higher quality of hip fracture care. - All quality indicators, as demanded by the two supervisory government agencies, i.e. the National Health Care Institute and the Health and Youth Care Inspectorate, can be calculated from the DHFA database. This prevents that physicians have to register the same data in multiple databases.

### Identification of factors influencing participation in the DHFA

To identify factors that may influence participation by medical professionals in the DHFA, the first author conducted semi-structured interviews with 8 trauma surgeons, 3 orthopaedic surgeons and 1 geriatrician/internist between 1 June 2017 and 8 November 2017. All the interviewees were members of at least one of the medical associations involved in the multidisciplinary DHFA.

The structure of the interviews was developed specifically for the DHFA according to the Theoretical Domains Framework [12, 13] (see Appendix 1). This framework was chosen, as determinants of behaviour related to both willingness and refusal to participate in the recently started clinical audit had to be identified [14]. The interviews were recorded and transcribed verbatim. The transcriptions were analysed according to the directed content approach, using the software package ATLAS.ti (ATLAS.ti Scientific Software Development GmBH, Berlin, Germany) [15]. First, possible factors that influence participation were highlighted in the transcripts, and secondly, the highlighted phrases were coded by two independent researchers. Since agreement of this double coding was sufficient in the first two transcripts (Cohen’s kappa of 0.89—see Appendix 2), the remaining 10 interviews were coded by one researcher (SV). In total, 76 factors that potentially influence participation in the DHFA were identified.

### Development and distribution of the questionnaire

The questionnaire aimed to explore which factors, identified in the semi-structured interviews, are actually independently associated with participation of hip fracture surgeons in the audit. As the surgeons are mainly responsible for the setup of the DHFA within hospitals, the questionnaire was not sent to geriatricians and internists. In the Netherlands, hip fracture surgery is performed by both orthopaedic surgeons and trauma surgeons. At the moment, the trauma surgeons treat the majority of hip fracture patients in the Netherlands, while orthopaedic surgeons operate relatively more patients who need a total hip replacement after a hip fracture.

The first part of the online questionnaire included questions covering the respondent characteristics: type of surgeon (orthopaedic or trauma surgeon), years of experience, annual number of hip fracture operations, type of hospital (academic or general hospital), familiarity with the DHFA (yes or no) and hospital level of participation in the DHFA. The level of participation was divided into four categories: not participating in the DHFA, and having no intention to do so; not participating in the DHFA, but intending to do so; partially participating in the DHFA with < 80% of the patients are registered; and actively participating with ≥ 80% of the patients are registered. If the respondent indicated in the first part not to be familiar with the audit, the second part of the questionnaire was not shown.

The second part of the questionnaire included the factors identified in the interviews that may influence participation in the DHFA. To limit the length of the questionnaire, the 76 factors were grouped into 21 overarching factors (Table 2). Henceforward, the word ‘factor’ refers to these 21 overarching factors. For each factor, the respondents could indicate their level of agreement on a 4-point Likert scale: totally disagree, partly disagree, partly agree and totally agree. All questions had to be filled out to complete the questionnaire.

**Table 2:** Characteristics of the 109 respondents divided by participation degree

	Total *N* (%)	Not participating in the DHFA, and not intending to do so *N* = 6 (5.5%)	Not participating in the DHFA, but intending to do so *N* = 29 (26.6%)	Partially participating in the DHFA, < 80% of patients registered *N* = 23 (21.1%)	Actively participating in the DHFA, ≥ 80% of patients registered *N* = 51 (46.8%)
Type of surgeon					
Orthopaedic surgeon	22 (20.2%)	1 (4.5%)	11 (50.0%)	3 (13.6%)	7 (31.8%)
Trauma surgeon	87 (79.8%)	5 (5.7%)	18 (20.7%)	20 (23.0%)	44 (50.6%)
Years of experience					
1–5 years	16 (14.7%)	2 (12.5%)	4 (25.0%)	1 (6.3%)	9 (56.3%)
6–10 years	33 (30.3%)	1 (3.0%)	10 (30.3%)	5 (15.2%)	17 (51.5%)
11–15 years	19 (17.4%)	0 (0.0%)	6 (31.6%)	4 (21.1%)	9 (47.4%)
> 15 years	41 (37.6%)	3 (7.3%)	9 (22.0%)	13 (31.7%)	16 (39%)
Type of hospital					
Academic hospital	15 (13.8%)	2 (13.3%)	8 (53.3%)	2 (13.3%)	3 (20.0%)
General hospital	94 (86.2%)	4 (4.3%)	21 (22.3%)	21 (22.3%)	48 (51.1%)
Annual number of operations					
1–20	19 (17.4%)	1 (5.3%)	10 (52.6%)	2 (10.5%)	6 (31.6%)
21–50	47 (43.1%)	2 (4.3%)	12 (25.5%)	10 (21.3%)	23 (48.9%)
51–100	32 (29.4%)	3 (9.4%)	3 (9.4%)	9 (28.1%)	17 (53.1%)
> 100	11 (10.1%)	0 (0.0%)	4 (36.4%)	2 (18.2%)	5 (45.5%)
Familiar with DHFA					
Yes	102 (93.6%)	2 (2.0%)	26 (25.5%)	23 (22.5%)	51 (50.0%)
No	7 (6.4%)	4 (57.1%)	3 (42.9%)	0 (0.0%)	0 (0.0%)

First, the questionnaire was distributed among the orthopaedic surgeons and trauma surgeons attending the annual national trauma congress in November 2017. After the congress, a link to the questionnaire was sent to all orthopaedic and trauma surgeons who are members of the Dutch Trauma Society, excluding those who had already completed the questionnaire. The targeted number of respondents was arbitrarily set at 100.

### Analysis

The data from the questionnaire was analysed using IBM SPSS Statistics® version 22. The respondent characteristics and the level of agreement to each of the 21 factors were reported using descriptive statistics. The categories for level of agreement were dichotomised for each factor into ‘disagree’ (combining the response categories ‘partly disagree’ and ‘totally disagree’) and ‘agree’ (combining ‘partly agree’ and ‘totally agree’), because for many factors, some categories were chosen by less than five respondents. For similar reasons, the four categories of participation were combined into two different dichotomous variables: the participation variable with ‘not participating’ versus ‘participating’ and the active participation variable with ‘partially participating with < 80% of patients registered’ versus ‘actively participating with ≥ 80% of patients registered’.

For both participation and active participation in the DHFA, the associations with respondent characteristics and the 21 overarching factors obtained from the interviews were first studied in a univariable logistic regression analysis. Next, respondent characteristics and factors found in the univariable analysis to be associated with participation or active participation (*p* < 0.10) were entered in a forward stepwise multivariable logistic regression analysis, with the factors/respondent characteristics as independent variables, and participation/active participation in the DHFA as dependent variables. In the multivariable model, predictors for participation or active participation were considered statistically significant if the *p* value was less than 0.05. Factors significantly increasing the participation in the DHFA were classified as facilitators, and factors significantly decreasing the participation in the DHFA as barriers.

## Results

At the national trauma congressin November 2017, 50 questionnaires were completed. Since the targeted number of respondents was at least 100, we sent out 257 questionnaires by mail after the congress.

In total (i.e. at the congress and by mail) 109 surgeons completed the questionnaire, when the questionnaires received at the congress and by mail were combined. Seven surgeons (four orthopaedic surgeons and three trauma surgeons) who completed the questionnaire did not know about the existence of the DHFA. The characteristics of the respondents across the different categories of participation are shown in Table 2. Our questionnaire was completed by 87 trauma surgeons (80%) and 22 orthopaedic surgeons (20%). Almost 14% of the respondents worked in an academic hospital and 86% in a general hospital.

### Factors most agreed on to influence participation in the DHFA

The three factors obtained from the interviews that were most agreed on in the questionnaire by both participants and non-participants concerned organisational aspects: ‘staffing capacity should be made available for the data collection in the DHFA (95.1%)’, ‘data import from the EHR into the DHFA should be automated (95.1%)’ and ‘participation in the DHFA should be supported financially by the hospital board (94.1%)’ (Table 3).

**Table 3:** Respondents’ agreement (partly/totally agree (versus partly/totally disagree)) with each factor for participation in the DHFA. The participating group is further stratified by participation degree

	Resondents' agreement with statement among respondents			
	Not participating in the DHFA *N* = 28	Participating in the DHFA *N* = 74	Degree of participation in the DHFA	
			< 80% of the patients registered *N* = 23	≥ 80% of the patients registered *N* = 51
1. At hospitals, staffing capacity must be made available for DHFA data collection.	27 (96.4%)	70 (94.6%)	22 (95.7%)	48 (94.1%)
2. Data entry into the DHFA from the electronic health record should be automated (registry at point of care).	26 (92.9%)	71 (95.9%)	23 (100.0%)	48 (94.1%)
3. Participation in the DHFA must be supported financially by the hospital board.	25 (89.3%)	71 (95.9%)	22 (95.7%)	49 (96.1%)
4. Implementation of the DHFA at hospital level requires a plan of action.	25 (89.3%)	69 (93.2%)	21 (91.3%)	48 (94.1%)
5. The DHFA increases the registration load for physicians.	27 (96.4%)	63 (85.1%)	20 (87.0%)	43 (84.3%)
6. The DHFA will provide insight into the actual quality of hip fracture care.	26 (92.9%)	63 (85.1%)	18 (78.3%)	45 (88.2%)
7. To ensure the proper organisation of the DHFA in hospitals, cooperation between the specialist areas involved (surgery, orthopaedics, geriatrics, internal medicine) is essential.	25 (89.3%)	65 (87.8%)	19 (82.6%)	46 (90.2%)
8. The DHFA is a tool for improving the quality of hip fracture care.	24 (85.7%)	62 (83.8%)	18 (78.3%)	44 (86.3%)
9. Too much data is requested in the DHFA.	22 (78.6%)	64 (86.5%)	20 (87.0%)	44 (86.3%)
10. The DHFA must do more than just give online feedback on outcomes.	23 (82.1%)	60 (81.1%)	20 (87.0%)	40 (78.4%)
11. The DHFA should be linked with other sources (municipal registries, Dutch Arthroplasty Register and Dutch Trauma Registry).	21 (75.0%)	64 (86.5%)	19 (82.6%)	45 (88.2%)
12. The added value of the DHFA lies in its being initiated and managed by medical practitioners themselves.	23 (82.1%)	55 (74.3%)	17 (73.9%)	38 (74.5%)
13. I am confident that the DHFA handles data with due care	21 (75.0%)	54 (73.0%)	15 (65.2%)	39 (76.5%)
14. The 3-month follow-up as required by the DHFA is not part of the standard clinical follow-up.	18 (64.3%)	59 (79.7%)	19 (82.6%)	40 (78.4%)
15. I am confident that the DHFA working group will make a proper assessment what data (quality indicators) can be made available to external parties.	19 (67.9%)	50 (67.7%)	12 (52.2%)	38 (74.5%)
16. The division of responsibilities for the execution of the DHFA between the specialists involved is not clear.	18 (64.3%)	49 (66.2%)	16 (69.6%)	33 (64.7%)
17. For the DHFA, a nationwide registry requirement should be introduced.	17 (60.7%)	51 (68.9%)	13 (56.5%)	38 (74.5%)
18. Data obtained from the DHFA offers relevant external parties (health insurers, National Health Care Institute) insight into the actual quality of hip fracture care.	17 (60.7%)	49 (66.2%)	11 (47.8%)	38 (74.5%)
19. The benefits of participation in the DHFA do not outweigh the costs.	15 (53.6%)	38 (51.4%)	15 (65.2%)	23 (45.1%)
20. The DHFA is going to lead to a cost reduction in hip fracture care.	14 (50.0%)	33 (44.6%)	11 (47.8%)	22 (43.1%)
21. The added value of the DHFA is not clear.	11 (39.3%)	35 (47.3%)	15 (65.2%)	20 (39.2%)

### Factors associated with actual participation in the DHFA

In the univariable analyses, none of the 21 overarching factors as identified in the interviews was significantly associated with actual participation in the DHFA (*p* > 0.10). Of the respondent characteristics, type of hospital (academic versus general hospital), type of surgeon (orthopaedic versus trauma) and annual number of hip operations had a univariable association with participation in the DHFA (*p* < 0.10) and were entered in the multivariable analysis (Table 4). Of those, working in an academic hospital (odds ratio [OR] 0.15, 95% confidence interval [CI] 0.04–0.52, *p* < 0.01) and being an orthopaedic surgeon (OR 0.30, 95% CI 0.10–0.90, *p* = 0.03) were independently associated with lower participation in the DHFA.

**Table 4:** Multivariable logistic regression analysis of participation in the DHFA, including factors with univariable *p* < 0.10

	Univariable OR (95% CI; *p*)	Multivariable OR (95% CI; *p*)
Type of surgeon (orthopaedic vs. trauma surgeon)	0.39 (0.14–1.12; 0.08)	0.30 (0.10–0.90; 0.03)
Type of hospital (academic vs. general)	0.18 (0.05–0.62; 0.01)	0.15 (0.04–0.52; < 0.01)
Annual number of operations		
1–20	Ref.	Ref.
21–50	4.58 (1.40–15.01; 0.01)	2.58 (0.59–11.26; 0.21)
51–100	6.50 (1.71–24.68; 0.01)	2.43 (0.45–13.19; 0.31)
> 100	2.19 (0.47–10.21; 0.32)	1.00 (0.17–5.79; 1.00)

Respondents not familiar with the DHFA were not shown the statements and are therefore excluded in this table

### Factors associated with active participation in the DHFA

Within the subgroup of surgeons who participated in the DHFA, three factors (‘the DHFA data provides insight into the actual quality of hip fracture care for relevant external parties’, ‘the DHFA working group makes a proper assessment what data can be made available to relevant external parties’ and ‘the added value of the DHFA is not clear’) were associated (*p* < 0.10) with active participation (i.e. registration of more than 80% of the hip fracture patients) in the univariable regression analyses and therefore entered in the multivariable analysis (Table 5). Of these, only the facilitator ‘the DHFA data provides insight into the actual quality of hip fracture care for external parties’ was significantly associated with active participation in the DHFA (OR 3.19, 95% CI 1.14–8.95, *p* = 0.03) in the multivariable regression analysis.

**Table 5:** Multivariable logistic regression analysis of participation degree (< 80% of the patients registered versus > 80% of the patients registered), including factors with univariable *p* < 0.10

	Univariable OR (95% CI; *p*)	Multivariable OR (95% CI; *p*)
Data obtained from the DHFA offers external parties (health insurers, National Health Care Institute) insight into the actual quality of hip fracture care.	3.19 (1.14–8.95; 0.03)	3.19 (1.14–8.95; 0.03)
I am confident that the DHFA working group makes a proper assessment what data (quality indicators) can be made available to external parties.	2.68 (0.95–7.52; 0.06)	1.55 (0.48–5.06; 0.47)
The added value of the DHFA is not clear.	0.34 (0.12–0.96; 0.04)	0.44 (0.15–1.28; 0.13)

Respondents not familiar with the DHFA have not been shown the statements and are therefore excluded in this

## Discussion

This study identified factors associated with hospital staff participation in a recently started nationwide clinical audit, the Dutch Hip Fracture Audit (DHFA). The most often agreed on factors influencing participation were related to the organisational context of the audit, i.e. how the data collection of the DHFA should be organised. However, only physician characteristics (type of hospital and type of surgeon), and no factors were independently associated with participation in the DHFA. For active participation (≥ 80% of patients registered), the opinion of surgeons that the DHFA data provides relevant external parties with insight into the actual quality of hip fracture care was significantly associated with the level of participation.

The factors identified through the semi-structured interviews are in line with the possible facilitators and barriers of a clinical audit previously described in literature [9, 10]. The additional value of our study is that it showed that none of these factors is actually associated with hospital participation, except that making data available to relevant external parties can serve as a facilitator for active participation.

### The organisational context of an audit

The primary goal of a clinical audit is to improve the quality of care. Similar to our results, most respondents in a survey among US surgeons also felt that an audit (the National Surgical Quality Improvement Program, NSQIP) would contribute to a better quality of care [16]. Interestingly, in the present study among both participants and non-participants, respondents agreed more on factors relating to the organisational context of the data collection than on the quality enhancement factors of an audit. At the start of the DHFA, no arrangements were made for the data collection and it was up to the hospital staff themselves to find a way to collect all the data for the audit. This might explain why the respondents in this study agreed more on the organisational factors of the audit rather than factors influencing its usefulness as a tool to improve the quality of patient care.

In literature, a previously reported facilitator for a successful audit is easy data collection, through the use of modern medical record systems [9]. A study of Cornish et al. showed that lack of support in data collection can also be a reason not to participate in an audit: in a survey among colorectal surgeons, the main reason not to participate in a 6-year running audit was the lack of technology support in the data submission [17]. Once an integrated audit has been established, minimal time and effort of a senior practitioner is needed [18].

As the factors most agreed on in the questionnaire were not specific to hip fracture management, our findings are applicable to upcoming clinical audits in general, regardless of their topic. Therefore, it is advisable that data collection is automated as much as possible and that time and resources are made available before the start of any audit. Hospitals in which the organisation of ongoing audits is well arranged can serve as examples for other hospitals where clinical auditing has yet to be implemented. Hospitals lacking the financial means to hire adequate staff for the DHFA data collection solved this by training of a research nurse in their employ, how to collect the data. They are now responsible for the data collection, but under the direct supervision of a surgeon in case of uncertainties.

### Factors that influence participation in an audit

Orthopaedic surgeons and trauma surgeons working in an academic hospital might be less likely to participate in the DHFA than orthopaedic surgeons and trauma surgeons working in a general hospital, respectively. This finding may be explained by the fact that in the Netherlands, orthopaedic and academic surgeons operate considerably lower numbers of hip fracture patients. For this reason, they might consider the effort of implementing an audit into the routine working procedures for relatively few patients per year not be worthwhile. This consideration is also true from a statistical point of view. Low-volume hospitals/wards are less interesting for benchmarking, as analyses for hospitals/wards with low patient volumes are underpowered to detect variation in outcomes of care [19]. One lesson to be learned from our findings, which we believe applies to every clinical audit, is that physicians treating low numbers of audit patients are less likely to participate in an audit. The questionnaire was only sent to surgeons, but it is most likely applicable for all physicians who treat a low number of audit patients.

### Factors that influence the participation degree

A facilitator for active (≥ 80% of patients registered) participation in an audit might be to make data available to relevant external parties (such as the National Health Care Institute and the healthcare insurance companies) and give them insight into the actual quality of hip fracture care by benchmarking hospitals. A possible explanation for this finding is that making hospital-specific data available soon after the start of an audit works as a trigger for hospitals to start registering, as they do not want to lag behind other hospitals. In the survey of Cornish et al., the national benchmarking facility also appeared to be the main reason to submit data to the bowel cancer audit [17]. Also, two other survey studies found that the general opinion of surgeons was that audit results should be publicly reported at hospital level [16, 20].

The optimal timing to make data available for relevant external parties is hard to define. From the second year of the DHFA on, the results of process indicators have been made available to the National Health Care Institute and the healthcare insurance companies. From the third year on, hospitals can choose to also make hospital-specific outcome indicators available, but from the fourth year onwards, this will be mandatory. The National Hip Fracture Database (NHFD) in the UK also made outcome indicators publicly transparent at hospital level in the third year after its start in 2007 [21].

In our opinion, it is important for all kinds of upcoming clinical audits to realise that data being available to external parties can be a determining factor for hospitals to participate actively in an audit. Therefore, it is advisable to agree at an early stage on how and when data will be disclosed to other relevant external parties.

### Lessons learned from other audits

Interventions in other audits that proved to be helpful in their implementation included financial rewards and obligatory participation. The need of financial support by the hospital board was also the third most agreed-on factor in our study. The NHFD has also shown that a financial reward, the Best Practice Tariff, can serve as a facilitator for hospitals to participate in a nationwide audit [22]. The Best Practice Tariff consists of six standards that have to be registered in the NHFD. If these standards are met, a hospital receives a financial reward [23].

Governmentally imposed participation has proven to facilitate participation. After the participation in the Dutch Surgical Colorectal Audit had become a quality indicator for the Health and Youth Care Inspectorate in the Netherlands, the participation rate rose to almost 100% [1, 2].

### Limitations

The grouping of the 76 factors identified in the semi-structured interviews into 21 overarching factors for the questionnaire has induced loss of information detail; it may have caused loss of the value of individual factors. The reason to group the factors was that, although we identified so many factors in the interviews, the questionnaire still should be clear and feasible and preferably be filled out within 10 min. We felt that sending out a questionnaire with all 76 factors would not render us the responses we needed. Another reason to group factors was from a statistical point of view: 76 factors and 5 respondent characteristics would induce the risk of multiple testing. Even with the 21 overarching factors this cannot be explained.

Another limitation is that the questionnaire was only sent to members of the Dutch Trauma Society, and therefore included only trauma and orthopaedic surgeons. Consequently, physicians from other medical disciplines such as geriatricians and internists were not invited to fill out the questionnaire. As a result, facilitators and barriers that are specific to non-surgeons were not included. This was a deliberate choice, since the setup of the DHFA data registry in a hospital is mainly the responsibility of the orthopaedic and trauma surgeons.

Regarding the representativeness of the respondents to the survey, the ratio between trauma surgeons and orthopaedic surgeons in our study (80/20) was comparable with that in the DHFA data for 2017 (71/29). However, the surgeons working in an academic hospital were overrepresented (14%) in the survey compared with the DHFA data for 2017 in which only 1% of the hip fracture patients were treated in an academic hospital. In addition, as responses were anonymous, possible clustering of respondents working at the same hospital may have occurred ,leaving unknown whether the respondents geographically represented the whole country. Both issues imply a possible selection bias.

Yet another limitation is that two of the three orthopaedic surgeons who were interviewed were not familiar with the DHFA. We may therefore have missed possible factors that are especially important for orthopaedic surgeons.

## Conclusion

We learned from this study that the data collection process of a nationwide clinical audit should either be performed by additional staff or be automated in order to avoid an increase in registration load for physicians. Physicians treating low numbers of audited patients are less likely to participate in an audit. Active participation in an audit may be promoted by offering relevant external parties, such as insurers, healthcare authorities or policymakers, insight into the actual quality of care. The use of these results will accommodate the successful implementation of new audits and the provided tools may offer opportunities to optimise participation in running audits.

## Appendix 1. Outline of the semi-structured interviews*.* The interviews were held in Dutch

**Table:**

Domains		Questions
1. Knowledge	1.1 Audit—general	- Are you familiar with clinical auditing? - What do you know about clinical auditing (do you believe it is evidence-based)? - Do you believe that auditing is a good tool for measuring/enhancing the quality of care? - Does your hospital already participate in any audit? ➔ If yes, what are the experiences? ➔ If not, why not?
	1.2 DHFA—specific	- Will a hip fracture audit make a useful contribution to hip fracture care? - Is this supported by the outcomes of existing hip fracture audits? - Are you familiar with the DHFA? ➔ If yes, what is the goal of the DHFA? How did you gain this knowledge? ➔ If not, are you a member of a professional association? Did you receive information about the DHFA from this association?
2. Skills		- Could you please explain how data is recorded in the DHFA? - Is this a reason for recording/not recording data? - Why?
3 Social/professional role and identity	3.1 Current	- How is hip fracture care organised at your hospital? Are care trajectories in place? - How is the cooperation with geriatrics set up? - Who delivers care at your department? Specialist, resident, nurse? - Do you work according to quality indicators/guidelines? - How does this impact on your participation in the DHFA?
	3.2 Future	- Will the DHFA affect hip fracture care at your hospital? - How would the outcomes affect your working method?
4. Beliefs about capabilities	4.1 Setup	- Did you manage to enter data into the DHFA? ➔ If yes, how did you go about this? ➔ If not, why not? - Would it be helpful if data entry/participation were simplified? - What action should be taken to enable participation in the DHFA?
	4.2 Continuation	- What action should be taken or has been taken to secure continued participation in the DHFA? - What action should be taken to promote more active participation in the DHFA? Why has such action not been taken so far?
5. Beliefs about consequences	5.1 Quality	- Do you expect the DHFA to contribute to higher-quality hip fracture care? - How will the DHFA affect the quality of hip fracture care?
	5.2 Costs	- What is the ultimate effect of the DHFA on costs?
	5.3 Influence	- What do you expect to be the outcomes of the DHFA for patients, professionals and hospitals?
6. Motivation and goals	6.1 Motivation	- Is the DHFA needed to measure quality? - Are alternatives available to enhance the quality of hip fracture care?
	6.2 Facilitators/barriers	- What are the facilitators of participation in the DHFA? - What are the reasons for refusing to participate in the DHFA or discontinuing participation?
7. Memory, attention and decision process	7.1 Own decision	- Given the current hospital-specific context, is it feasible for you to take part in the DHFA? ➔If yes, how did you secure this? ➔If not, what should change to make participation feasible?
	7.2 Parties involved	- What persons at your hospital are involved in the decision whether or not to participate in the DHFA? - How do they influence the decision-making process? - If they are not in favour of participation, what has been done or should be done to secure their support for participation in the DHFA?
8 Environmental context and resources	8.1 Organisation	- To what extent do environmental factors (nationwide, regional, executive, organisational level) have an impact on participation or non-participation in the DHFA? - How does the multidisciplinary nature of the DHFA influence the participation? - What considerations may lead to participation or non-participation?
	8.2 Time	- How much time do you expect to be needed to participate in the DHFA? - Will this influence your decision whether or not to participate in the DHFA?
	8.3 Financial	- What costs will be involved in initiating/continuing the DHFA? - Will financial considerations influence your decision as to participation in the DHFA? - How does this impact on your participation in the DHFA?
9. Social influences	9.1 Professionals	- Do professionals of the same medical specialty (partnership/regional/nationwide) influence participation? - Do professionals of a different medical specialty (e.g. surgery, orthopaedics, internal medicine, geriatrics) influence participation?
	9.2 Patients	- Does the patient category influence the decision whether or not participate in the DHFA?
10. Emotion		- What consequences may the outcomes of the DHFA have? - How does this impact on your participation in the DHFA? - What action should be taken to remove or encourage these factors? - Can the outcomes be used to identify underperformers? And does this influence your participation?
11. Behavioural regulation	11.1 Personal	- What should be done on both personal and organisational levels to enable participation in the DHFA? - How can the introduction of the DHFA be supported on both personal and organisational levels?
	11.2 Organisational	- What would the consequences be if recording data in the DHFA were made compulsory? - What is your opinion as quality assessment is based on quality indicators emerging from the DHFA?
12. Nature of the behaviours	12.1 Current action	- Who is supposed to do what and when to enable participation in the DHFA? - How long will it take until all persons know what is expected from?
	12.2 Future action	- How can the implementation within hospitals be speeded up? - How can be secured that all parties involved stay motivated to participate?

## Appendix 2. Cohen’s kappa calculation. Identified versus non-identified barriers/facilitators

**Table:**

		Coder 2		
		Identified	Not identified	
Coder 1	Identified	47	1	48
	Not identified	2	0	2
		49	1	50

Chance frequency cell A = (49 × 48)/50 = 47.04

Chance frequency cell D = (1 × 2)/50 = 0.04

Chance agreement = (47.04 + 0.04)/100 = 0.47

Chance corrected observed agreement = (47/50) − 0.47 = 0.47 (47%)

Chance corrected potential agreement = 100% − chance agreement (0.47) = 0.53 (53%)

Cohen’s kappa = (% chance corrected observed agreement/% chance corrected potential agreement) 47%/53% = 0.89
